# Supplementary material for: Impact of Tralopyril and Triazolyl Glycosylated Chalcone in Human Retinal Cells’ Lipidome
Source: Molecules. 2022 Aug 17;27(16):5247. doi: 10.3390/molecules27165247 (PMC9412578; doi:10.3390/molecules27165247)
Supplement: Supplementary file 1 [file molecules-27-05247-s001.zip › molecules-1723805-supplementary.pdf]

Supplementary Materials

Impact of tralopyril and triazolyl glycosylated chalcone in human retinal cells lipidome

Cátia Vilas-Boas [1,2], Logan Running [3], Daniela Pereira [1,2], Honorina Cidade [1,2], Marta Correia-da-Silva [1,2], Gunes Ekin Atilla-Gokcumen [3,*] and Diana S. Aga [3,*]

1 Laboratory of Organic and Pharmaceutical Chemistry, Department of Chemical Sciences, Faculty of Pharmacy, University of Porto, 4050-313 Porto, Portugal
2 CIIMAR/CIMAR—Interdisciplinary Center for Marine and Environmental Research, University of Porto, 4450-208 Matosinhos, Portugal
3 Chemistry Department, University at Buffalo, The State University of New York, Buffalo, NY 14260, USA
* Correspondence: ekinatil@buffalo.edu (G.E.A.-G.); dianaaga@buffalo.edu (D.S.A.)

Table of contents

1. Synthesis and purity of compound 1...…………....……………………...……………... 3

2. Target lipidomic.………..………………………………………………………………….4

3. References………………...…………………………………………………….……..11

1. Purity of compound 1

A promising nature-inspired AF compound, a triazolyl glycosylated chalcone (compound 1) was obtained by click chemistry according to our previously described synthesis [1]. Its purity was evaluated by a high-performance liquid chromatography method coupled to an ultraviolet detector (HPLC-UV) (Figure S1).

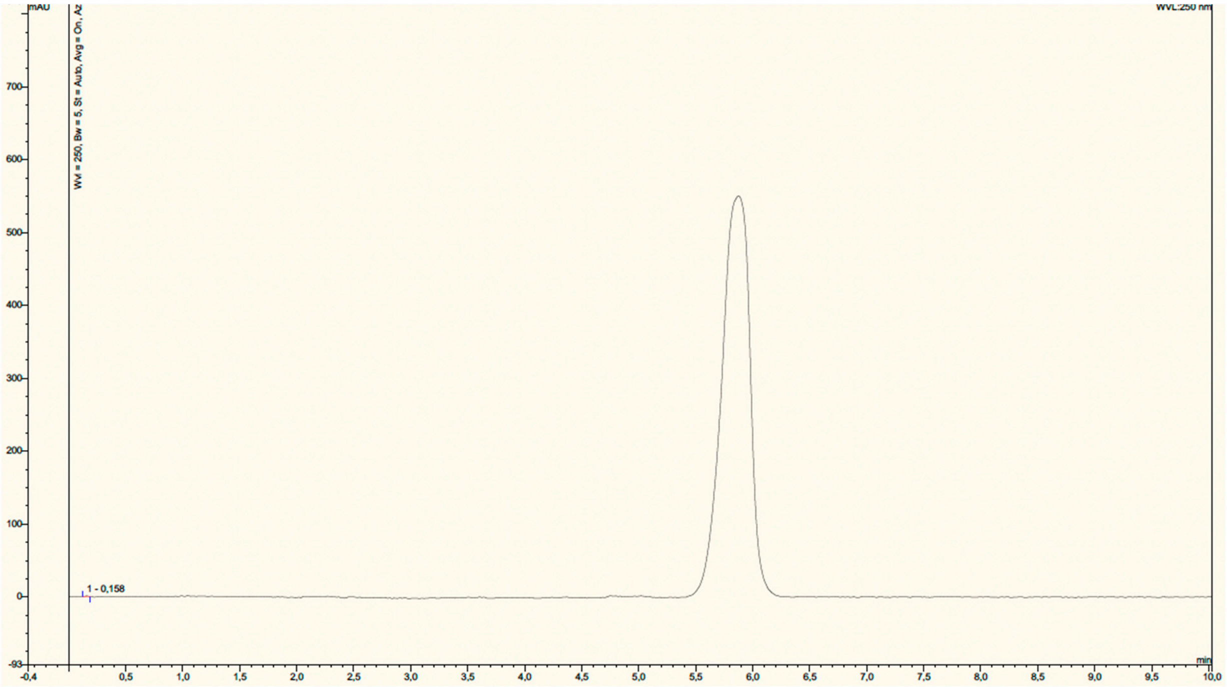

Figure S1. Representative chromatograms of pure compound **1** (200 μM) dissolved in MeOH with a chromatographic signal at 5.9 min at 250 nm when a mobile phase containing water/acetonitrile (30:70 (v/v)) acidified with acetic acid (pH 2.5) was used.

2. Lipidomics results

Table S1. Lipid standards used, mass-to-charge ratios (*m/z*'s), retention time (RT) of internal standards. The total carbon number on the acyl chains and the degree of unsaturation of the fatty acyl chains in the lipid species are listed, i.e. 16:0 corresponds to 16 carbons and 0 double bonds.

						Compound 1		Econea®	
**Species**	**Adduct**	***m/z***	**PPM error**	**RT (Min)**	**Abundance**	**Fold Change**	**P-value**	**Fold Change**	**P-Value**
D9 Oleic acid	$[M - H]^-$	290.3051	-5.17	36.897	6.57E+05	1.07	0.2071	1.05	0.6305
C17:0 CER	$[M - H]^-$	550.5205	-6.36	64.339	9.89E+07	1.05	0.2440	1.02	0.8000
C39:0 TAG	$[M + H]^+$	698.6293	2.58	64.126	7.72E+07	1.08	0.1983	1.03	0.7998
C57:0 TAG	$[M + H]^+$	950.9110	-5.99	69.461	3.39E+07	0.89	0.0691	1.00	0.9307
D70 DSPC	$[M + H]^+$	861.0724	3.60	59.771	7.02E+07	1.00	0.9667	0.92	0.2989
C17:0 Sphingosine	$[M + H]^+$	286.2700	8.73	38.794	1.77E+08	1.14	0.1689	0.88	0.4806
C17:0 SM	$[M + H]^+$	717.5905	3.07	56.696	7.20E+07	1.00	0.8917	1.18	0.0740
C17:0 Glucosoceramide	$[M + H]^+$	696.5773	1.29	57.128	2.22E+08	1.07	0.2071	1.05	0.6305

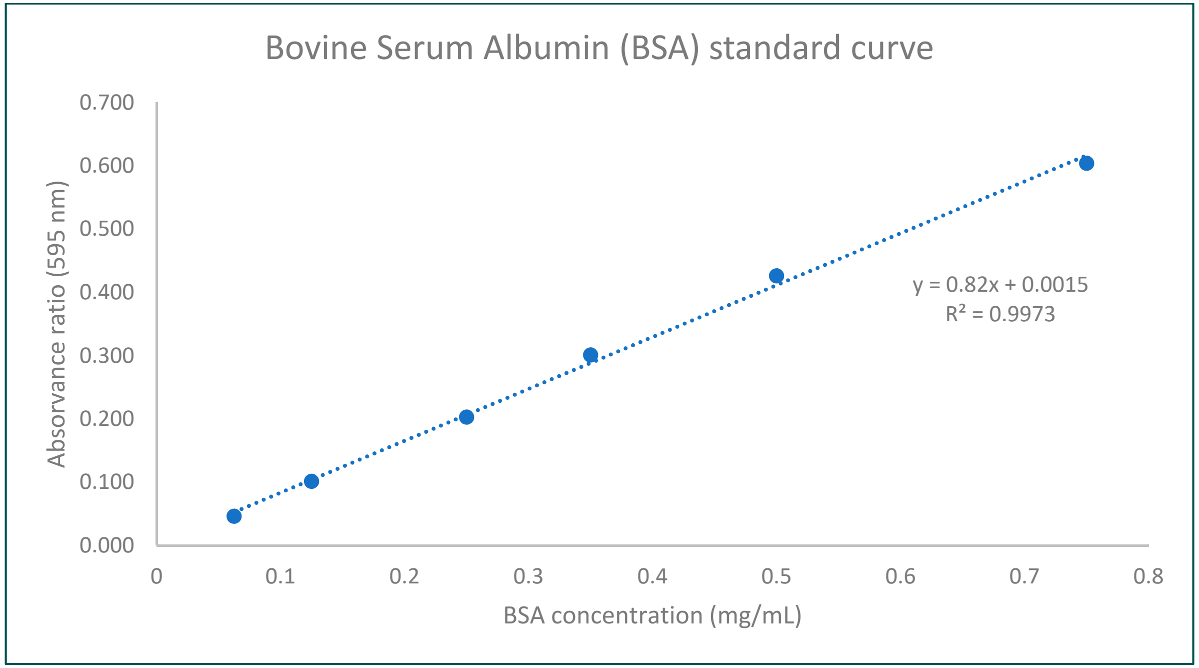

Figure S2. Linearity of the Bradford calibration graph.

Table S2. Protein normalization using Bradford protein assay. Cell extracts were reconstituted so that each sample was normalized to a concentration relative to the replicate with the lowest protein content. Due to cell toxicity, there was lower protein content in the Econea® treated samples. To account for this, aliquots of each control group was diluted to normalize protein content in the controls to the Econea® treated samples. Protein normalization for Compound 1 and Econea® with their respective controls are seen here.

Samples	Amount of protein per sample (μg) (in pellet collected, prior to normalization)	Protein concentration in μg/μL (10 μL injected for analysis)	Protein content of each sample analysed in μg(after normalization)
Control	228.3	1.46	14.6
	188.1		
	175.5		
Compound 1	276.4		
	237.8		
	209.4		
Diluted Control for Econea®	93.8	0.6	6
	77.3		
	72.1		
Econea®	101.3		
	85.6		
	73		

Table S3. Raw abundances for controls and treated samples (N=3).

Species	Approx RT (Min)	*m/z*	Control 1	Control 2	Control 3	Compound 1	Compound 2	Compound 3	Diluted Control 1	Diluted Control 2	Diluted Control 3	Econea 1	Econea 2	Econea 3

PC C28:3	38.7	672.4604	3.47E+08	3.91E+08	2.68E+08	2.31E+08	3.71E+08	3.80E+08	2.11E+08	2.29E+08	1.54E+08	3.11E+08	3.53E+08	3.16E+08
PC C30:1	55.2	704.5230	1.07E+08	1.25E+08	1.47E+08	8.30E+07	9.58E+07	7.91E+07	4.56E+07	4.39E+07	5.51E+07	1.02E+08	1.08E+08	1.49E+08
PC C30:0	56.0	706.5387	3.48E+08	4.00E+08	4.53E+08	3.41E+08	3.81E+08	3.41E+08	1.66E+08	1.64E+08	1.93E+08	2.19E+08	2.30E+08	2.84E+08
PC C32:3	55.9	728.5230	5.59E+07	7.00E+07	8.37E+07	5.63E+07	6.32E+07	5.34E+07	2.10E+07	1.97E+07	2.68E+07	5.58E+07	5.86E+07	7.87E+07
PC C32:2	55.9	730.5387	1.89E+08	2.21E+08	2.59E+08	1.62E+08	1.90E+08	1.63E+08	8.60E+07	8.07E+07	1.01E+08	1.75E+08	1.90E+08	2.43E+08
PC C32:1	56.7	732.5543	7.54E+08	8.20E+08	8.81E+08	7.57E+08	8.39E+08	7.38E+08	4.37E+08	4.12E+08	4.79E+08	6.55E+08	6.72E+08	7.96E+08
PC 32:0	57.4	734.5700	3.92E+08	4.06E+08	4.34E+08	3.44E+08	3.38E+08	3.34E+08	2.58E+08	2.57E+08	2.84E+08	1.85E+08	1.89E+08	2.05E+08
PC C34:4	56.7	754.5387	3.23E+07	4.35E+07	4.53E+07	4.36E+07	4.10E+07	3.91E+07	1.95E+07	1.91E+07	2.34E+07	2.33E+07	2.49E+07	3.13E+07
PC C34:3	56.3	756.5543	1.57E+08	1.69E+08	1.90E+08	1.35E+08	1.47E+08	1.30E+08	8.71E+07	8.55E+07	9.92E+07	1.49E+08	1.51E+08	1.89E+08
PC C34:2	57.1	758.5700	6.93E+08	7.26E+08	7.77E+08	6.51E+08	6.87E+08	6.21E+08	4.45E+08	4.19E+08	4.80E+08	6.26E+08	6.27E+08	7.25E+08
PC C34:1	57.4	760.5856	1.09E+09	1.11E+09	1.12E+09	1.13E+09	1.03E+09	1.10E+09	8.95E+08	8.62E+08	9.06E+08	8.95E+08	9.02E+08	9.72E+08
PC C34:0	58.8	762.6013	8.74E+07	8.99E+07	9.85E+07	1.01E+08	1.05E+08	9.40E+07	5.45E+07	5.34E+07	6.02E+07	3.33E+07	3.27E+07	3.60E+07
PC C36:4	57.4	782.5700	3.16E+08	3.76E+08	3.70E+08	3.18E+08	3.40E+08	3.16E+08	2.93E+08	2.58E+08	2.77E+08	3.31E+08	3.40E+08	3.66E+08
PC C36:3	57.6	784.5856	3.33E+08	3.46E+08	3.56E+08	3.25E+08	3.43E+08	3.12E+08	2.32E+08	2.12E+08	2.33E+08	3.08E+08	3.08E+08	3.48E+08
PC C36:2	58.4	786.6013	7.86E+08	8.08E+08	8.29E+08	7.82E+08	7.99E+08	7.50E+08	5.83E+08	5.37E+08	5.91E+08	6.60E+08	6.51E+08	7.22E+08
PC C36:1	59.3	788.6169	4.77E+08	5.06E+08	5.30E+08	4.64E+08	4.86E+08	4.40E+08	2.97E+08	2.63E+08	3.31E+08	2.95E+08	2.97E+08	3.43E+08
PC C38:5	57.7	808.5856	3.63E+08	3.89E+08	3.87E+08	2.92E+08	3.11E+08	2.92E+08	2.71E+08	2.55E+08	2.93E+08	3.26E+08	3.28E+08	3.65E+08
PC C38:4	58.8	810.6013	3.93E+08	4.09E+08	4.26E+08	3.07E+08	3.36E+08	3.14E+08	2.72E+08	2.57E+08	2.69E+08	2.96E+08	3.07E+08	3.47E+08
PC C38:3	59.0	812.6169	2.08E+08	2.13E+08	2.31E+08	1.61E+08	1.73E+08	1.60E+08	1.29E+08	1.23E+08	1.44E+08	1.18E+08	1.22E+08	1.41E+08
PC C38:2	59.5	814.6326	4.04E+07	4.19E+07	4.72E+07	4.06E+07	4.23E+07	3.91E+07	2.26E+07	2.10E+07	2.59E+07	2.05E+07	2.06E+07	2.59E+07
PC C40:6	58.7	834.6013	8.20E+07	8.96E+07	9.67E+07	6.66E+07	6.97E+07	6.39E+07	5.31E+07	5.09E+07	6.09E+07	3.33E+07	3.35E+07	3.77E+07
PC C40:5	58.9	836.6169	1.59E+08	1.69E+08	1.81E+08	1.11E+08	1.14E+08	1.10E+08	1.04E+08	9.43E+07	1.15E+08	9.46E+07	9.55E+07	1.08E+08

PC C40:4	59.5	838.6326	6.08E+07	6.33E+07	7.40E+07	5.05E+07	5.10E+07	5.10E+07	3.26E+07	2.86E+07	4.12E+07	3.94E+07	3.75E+07	4.59E+07
PC C40:2	60.7	842.6639	3.53E+06	3.68E+06	4.49E+06	3.86E+06	4.00E+06	4.04E+06	1.57E+06	1.69E+06	2.12E+06	2.14E+06	1.89E+06	2.56E+06
PC C40:1	61.5	844.6795	2.39E+06	2.71E+06	3.16E+06	2.94E+06	3.04E+06	3.10E+06	1.35E+06	1.30E+06	1.37E+06	7.78E+05	6.48E+05	8.04E+05
PC C40:0	62.4	846.6952	4.91E+05	4.80E+05	6.21E+05	8.96E+05	8.97E+05	9.35E+05	3.68E+05	3.70E+04	3.43E+05	2.27E+05	3.13E+05	2.43E+05
PC C42:6	59.0	862.6326	1.30E+07	1.53E+07	1.48E+07	8.23E+06	7.82E+06	8.43E+06	8.43E+06	7.61E+06	8.89E+06	5.87E+06	5.67E+06	8.00E+06
PC C42:5	59.7	864.6482	2.69E+07	2.87E+07	3.12E+07	1.71E+07	1.91E+07	1.79E+07	1.47E+07	1.37E+07	1.60E+07	1.22E+07	1.19E+07	1.53E+07
PC C42:4	60.4	866.6639	1.12E+07	1.09E+07	1.24E+07	6.09E+06	6.85E+06	6.63E+06	4.84E+06	4.62E+06	5.28E+06	3.74E+06	3.74E+06	4.83E+06
PC C42:3	61.1	868.6795	3.19E+06	3.54E+06	4.05E+06	2.67E+06	2.71E+06	2.58E+06	2.31E+06	1.71E+06	1.94E+06	9.39E+05	8.08E+05	9.40E+05
PC C42:2	61.8	870.6952	3.93E+06	4.69E+06	5.16E+06	4.55E+06	4.85E+06	4.66E+06	2.32E+06	2.22E+06	2.37E+06	1.14E+06	1.09E+06	1.44E+06
PC C42:1	62.8	872.7108	3.92E+06	4.45E+06	4.86E+06	5.29E+06	5.85E+06	6.03E+06	2.01E+06	1.99E+06	2.30E+06	9.12E+05	8.94E+05	1.22E+06
PC C42:0	63.4	874.7265	1.49E+06	1.66E+06	1.82E+06	2.65E+06	3.08E+06	2.98E+06	8.69E+05	8.40E+05	9.19E+05	4.89E+05	4.64E+05	4.74E+05
PC C44:6	60.1	890.6639	8.59E+06	9.45E+06	1.06E+07	4.45E+06	5.14E+06	5.07E+06	3.86E+06	3.95E+06	4.12E+06	2.35E+06	2.36E+06	2.75E+06
PC C44:5	60.7	892.6795	4.16E+06	4.80E+06	5.13E+06	2.18E+06	2.18E+06	2.10E+06	2.33E+06	2.18E+06	2.30E+06	1.28E+06	1.09E+06	1.21E+06
PC C44:4	61.4	894.6952	3.95E+06	4.90E+06	5.45E+06	2.18E+06	2.92E+06	2.64E+06	2.25E+06	2.08E+06	2.46E+06	1.10E+06	1.04E+06	1.44E+06
PC C44:3	62.1	896.7108	2.10E+06	2.62E+06	2.21E+06	1.83E+06	2.44E+06	1.95E+06	1.52E+06	1.20E+06	1.61E+06	4.37E+05	4.72E+05	6.35E+05
PC C44:2	62.8	898.7265	5.30E+06	6.25E+06	6.80E+06	6.19E+06	6.92E+06	6.87E+06	3.27E+06	2.70E+06	2.66E+06	9.62E+05	9.69E+05	1.10E+06
PC C44:1	63.7	900.7421	3.74E+06	4.20E+06	4.70E+06	6.86E+06	7.27E+06	6.68E+06	1.94E+06	1.83E+06	2.12E+06	9.49E+05	9.68E+05	1.09E+06
PC C44:0	64.2	902.7578	3.73E+05	3.65E+05	4.12E+05	6.89E+05	8.37E+05	6.38E+05	2.33E+05	2.00E+05	2.98E+05	2.23E+05	1.98E+05	2.57E+05
PC C46:6	61.1	918.6952	3.02E+06	3.26E+06	4.05E+06	1.99E+06	2.41E+06	2.10E+06	1.69E+06	1.45E+06	1.87E+06	8.54E+05	8.71E+05	9.41E+05
PC C46:5	62.5	920.7108	2.66E+06	2.69E+06	3.40E+06	2.03E+06	2.51E+06	2.57E+06	1.40E+06	1.31E+06	1.64E+06	6.74E+05	7.64E+05	7.98E+05
PC C46:4	63.4	922.7265	1.87E+06	2.06E+06	2.55E+06	2.04E+06	2.16E+06	2.23E+06	1.05E+06	1.06E+06	1.12E+06	6.01E+05	5.00E+05	6.53E+05
PC C46:3	63.4	924.7421	1.26E+06	1.71E+06	1.97E+06	1.70E+06	1.91E+06	1.75E+06	8.01E+05	7.19E+05	8.29E+05	3.89E+05	3.60E+05	3.40E+05
PC C46:2	63.8	926.7578	1.33E+06	1.35E+06	1.49E+06	2.00E+06	1.94E+06	1.83E+06	6.51E+05	5.16E+05	6.33E+05	2.89E+05	2.30E+05	2.88E+05

PC C48:6	63.3	946.7265	3.82E+05	4.37E+05	3.85E+05	6.05E+05	6.59E+05	6.93E+05	1.78E+05	2.25E+05	1.73E+05	ND	ND	ND
PC C48:5	63.5	948.7421	8.37E+05	8.39E+05	9.80E+05	8.52E+05	7.61E+05	7.22E+05	4.28E+05	3.96E+05	4.79E+05	2.74E+05	3.37E+05	2.44E+05
DAG C34:2	64.5	575.5039	1.17E+06	1.44E+06	5.20E+05	1.42E+06	1.39E+06	1.02E+06	7.64E+05	7.35E+05	8.06E+05	7.37E+05	8.15E+05	3.51E+05
DAG C34:1	64.9	577.5196	3.32E+06	4.25E+06	9.02E+05	3.16E+06	4.81E+06	4.43E+06	1.29E+06	1.92E+06	2.51E+06	1.93E+06	2.06E+06	4.79E+05
DAG C36:4	64.6	599.5039	1.02E+06	1.18E+06	3.87E+05	7.32E+05	1.11E+06	1.18E+06	4.05E+05	5.43E+05	6.71E+05	5.49E+05	6.12E+05	2.04E+05
DAG C36:2	64.5	603.5352	1.14E+07	1.27E+07	5.11E+06	7.66E+06	1.20E+07	1.22E+07	5.21E+06	6.52E+06	7.40E+06	6.14E+06	6.50E+06	2.39E+06
DAG C36:1	64.9	605.5509	1.42E+07	1.68E+07	4.36E+06	1.10E+07	1.64E+07	1.65E+07	5.36E+06	7.24E+06	9.28E+06	8.04E+06	8.29E+06	2.47E+06
DAG C38:5	64.8	625.5196	2.80E+06	4.26E+06	8.38E+05	2.29E+06	3.58E+06	3.68E+06	1.14E+06	1.50E+06	2.04E+06	1.57E+06	1.98E+06	3.12E+05
DAG C38:4	65.2	627.5352	1.37E+07	1.70E+07	5.24E+06	9.99E+06	1.57E+07	1.45E+07	6.11E+06	7.22E+06	9.17E+06	7.39E+06	8.47E+06	2.30E+06
DAG C36:4	60.8	617.5145	7.44E+06	7.16E+06	7.22E+06	8.60E+06	6.65E+06	5.18E+06	6.05E+06	6.41E+06	6.77E+06	4.73E+06	3.68E+06	4.37E+06
DAG C36:0	61.3	643.5302	5.71E+06	5.84E+06	5.44E+06	5.02E+06	4.27E+06	3.51E+06	3.54E+06	3.54E+06	4.00E+06	3.44E+06	2.69E+06	2.71E+06
TAG C30:4	56.2	564.4259	7.45E+05	7.10E+05	7.03E+05	9.50E+05	9.89E+05	9.22E+05	5.32E+05	5.49E+05	5.53E+05	1.01E+06	1.23E+06	7.38E+05
TAG C38:4	54.4	676.5511	8.54E+06	1.08E+07	1.39E+07	6.56E+06	8.72E+06	7.37E+06	2.32E+06	3.11E+06	4.03E+06	2.55E+06	3.10E+06	4.01E+06
TAG C48:4	61.5	816.7076	2.72E+07	3.30E+07	3.92E+07	1.88E+07	2.29E+07	2.40E+07	9.39E+06	1.18E+07	1.43E+07	7.06E+06	8.19E+06	9.43E+06
TAG C50:4	66.9	848.7702	2.61E+07	3.03E+07	1.83E+07	3.39E+07	3.42E+07	2.89E+07	1.65E+07	1.83E+07	1.61E+07	1.61E+06	1.69E+07	1.84E+07
TAG C50:2	66.9	848.7707	1.74E+07	1.98E+07	2.12E+07	2.48E+07	2.41E+07	2.13E+07	1.12E+07	1.07E+07	1.17E+07	1.01E+07	9.59E+06	1.08E+07
TAG C50:1	67.2	850.7864	1.74E+07	2.04E+07	2.31E+07	2.54E+07	2.89E+07	2.80E+07	1.32E+07	1.20E+07	1.32E+07	9.27E+06	8.71E+06	8.87E+06
TAG C54:5	66.8	898.7864	2.30E+07	2.01E+07	2.24E+07	1.81E+07	1.80E+07	1.38E+07	1.67E+07	1.69E+07	1.69E+07	2.66E+07	2.43E+07	2.58E+07
TAG C54:4	67.1	900.8020	1.03E+07	8.16E+06	7.86E+06	1.24E+07	1.09E+07	7.90E+06	4.46E+06	3.36E+06	3.67E+06	5.76E+06	5.27E+06	6.07E+06
TAG C54:1	67.7	906.8490	7.01E+05	5.96E+05	6.48E+05	2.03E+06	1.90E+06	1.81E+06	3.40E+05	3.40E+05	3.86E+05	3.30E+05	3.16E+05	4.25E+05
TAG C54:0	68.7	908.8646	1.35E+06	1.29E+06	1.33E+06	1.65E+06	1.87E+06	1.34E+06	1.49E+06	1.50E+06	1.35E+06	1.52E+06	1.41E+06	1.29E+06
TAG C56:8	66.5	920.7707	2.42E+06	2.83E+06	2.77E+06	1.11E+06	1.04E+06	8.15E+05	1.17E+06	8.98E+05	9.72E+05	3.01E+06	2.73E+06	3.38E+06
TAG C56:6	67.0	924.8020	9.97E+06	1.15E+07	1.23E+07	7.48E+06	8.63E+06	7.38E+06	8.28E+06	9.08E+06	9.37E+06	1.79E+07	1.55E+07	1.82E+07

TAG C58:8	67.0	948.8020	5.55E+06	6.57E+06	3.35E+06	3.53E+06	3.57E+06	2.56E+06	3.35E+06	2.67E+06	3.38E+06	8.88E+06	8.61E+06	1.12E+07
TAG C58:2	69.0	960.8959	9.18E+05	8.40E+05	9.79E+05	1.86E+06	1.44E+06	1.33E+06	3.34E+05	2.98E+05	3.45E+05	3.55E+05	2.53E+05	3.29E+05
TAG C60:6	67.8	980.8646	4.98E+06	4.79E+06	4.59E+06	6.82E+06	6.12E+06	5.47E+06	5.81E+06	4.57E+06	4.82E+06	5.46E+06	4.59E+06	4.89E+06
TAG C60:4	68.6	984.8959	4.06E+05	3.54E+05	4.71E+05	5.23E+05	5.45E+05	4.67E+05	2.90E+05	3.51E+05	4.21E+05	1.28E+05	1.25E+05	1.25E+05
Cer C14:0	61.7	508.4730	8.76E+05	9.52E+05	8.98E+05	5.05E+05	4.90E+05	4.11E+05	2.99E+05	2.72E+05	2.36E+05	4.76E+05	6.82E+05	7.58E+05
Cer C16:1	62.2	534.4886	9.51E+05	8.82E+05	9.27E+05	6.19E+05	5.35E+05	4.07E+05	3.76E+05	3.24E+05	2.52E+05	2.75E+05	3.64E+05	3.62E+05
Cer C18:1	64.1	562.5199	3.16E+05	3.05E+05	3.06E+05	1.29E+05	1.40E+05	1.06E+05	1.19E+05	1.22E+05	9.84E+04	4.11E+04	3.17E+04	3.43E+04
Cer C18:0	65.1	564.5356	3.09E+06	3.32E+06	3.30E+06	1.38E+06	1.50E+06	1.34E+06	1.15E+06	1.29E+06	1.13E+06	6.51E+05	8.27E+05	8.34E+05
Cer C20:0	66.6	592.5669	1.30E+06	1.36E+06	1.40E+06	6.09E+05	6.86E+05	5.76E+05	6.15E+05	5.48E+05	5.34E+05	2.95E+05	3.26E+05	3.79E+05
Cer C22:1	66.6	618.5825	1.91E+06	1.95E+06	2.12E+06	8.58E+05	8.85E+05	8.40E+05	1.03E+06	9.04E+05	8.73E+05	2.51E+05	2.99E+05	3.51E+05
Cer C22:0	67.8	620.5982	6.75E+06	7.10E+06	7.38E+06	4.26E+06	4.48E+06	4.02E+06	3.09E+06	2.86E+06	2.63E+06	1.19E+06	1.50E+06	1.67E+06
Cer C24:1	67.8	646.6138	1.64E+07	1.68E+07	1.76E+07	1.02E+07	1.08E+07	9.28E+06	7.65E+06	7.33E+06	6.48E+06	3.26E+06	4.08E+06	4.53E+06
Cer C24:0	69.2	648.6295	1.87E+07	2.00E+07	1.98E+07	1.99E+07	2.16E+07	1.92E+07	7.91E+06	7.49E+06	6.83E+06	2.10E+06	2.68E+06	3.05E+06
Cer C26:1	69.2	674.6451	2.76E+06	2.75E+06	3.05E+06	2.07E+06	2.10E+06	1.93E+06	9.70E+05	1.05E+06	9.34E+05	4.93E+05	6.25E+05	6.50E+05
Cer C26:0	71.0	676.6608	1.76E+06	2.08E+06	2.12E+06	2.71E+06	2.79E+06	2.42E+06	7.27E+05	7.50E+05	6.61E+05	2.71E+05	2.92E+05	3.22E+05
DiHCer C16:0	64.1	538.5199	1.03E+06	1.05E+06	1.02E+06	4.75E+05	4.93E+05	4.60E+05	4.12E+05	4.08E+05	3.95E+05	2.86E+06	3.68E+06	4.25E+06
DiHCer C18:0	65.6	566.5512	4.90E+05	6.09E+05	5.88E+05	2.85E+05	2.68E+05	2.69E+05	2.63E+05	3.08E+05	2.05E+05	7.45E+05	8.58E+05	9.54E+05
DiHCer C20:0	67.0	594.5825	5.29E+05	4.85E+05	4.83E+05	2.57E+05	3.18E+05	2.13E+05	2.58E+05	2.64E+05	2.20E+05	4.20E+05	4.38E+05	4.35E+05
DiHCer C22:0	68.3	622.6138	1.56E+06	1.73E+06	1.64E+06	8.93E+05	1.06E+06	9.18E+05	8.20E+05	7.34E+05	7.34E+05	1.25E+06	1.21E+06	1.32E+06
DiHCer C24:0	69.2	650.6451	3.34E+06	3.47E+06	3.65E+06	3.06E+06	3.61E+06	3.01E+06	1.60E+06	1.43E+06	1.48E+06	1.11E+06	1.22E+06	1.34E+06
SM C14:0	54.4	675.5436	2.25E+07	2.88E+07	3.64E+07	1.85E+07	2.38E+07	2.05E+07	6.68E+06	8.21E+06	1.17E+07	1.01E+07	1.18E+07	1.40E+07
SM C16:1	55.0	701.5592	5.41E+07	6.64E+07	8.35E+07	4.58E+07	5.75E+07	4.92E+07	1.53E+07	1.89E+07	2.54E+07	1.75E+07	1.98E+07	2.69E+07
SM C16:0	55.9	703.5749	2.93E+08	3.52E+08	4.24E+08	2.47E+08	3.35E+08	2.74E+08	8.71E+07	1.14E+08	1.45E+08	1.13E+08	1.36E+08	1.67E+08

SM C24:0	61.5	815.7001	4.84E+07	5.84E+07	6.99E+07	3.37E+07	4.06E+07	4.28E+07	1.69E+07	2.13E+07	2.59E+07	1.31E+07	1.49E+07	1.69E+07
SM C18:1	56.6	729.5905	3.73E+06	4.37E+06	5.15E+06	2.49E+06	2.89E+06	2.32E+06	1.29E+06	1.54E+06	2.14E+06	1.46E+06	1.59E+06	1.75E+06
SM C18:0	57.5	731.6062	1.13E+07	1.39E+07	1.50E+07	7.61E+06	9.55E+06	8.26E+06	4.46E+06	5.98E+06	7.45E+06	4.62E+06	5.60E+06	5.47E+06
SM C22:0	60.3	787.6688	2.55E+07	3.10E+07	3.78E+07	1.43E+07	1.87E+07	1.54E+07	7.06E+06	9.85E+06	1.22E+07	5.70E+06	7.73E+06	8.47E+06
SM C24:1	60.3	813.6844	7.85E+07	8.95E+07	1.06E+08	4.35E+07	5.46E+07	5.15E+07	2.64E+07	3.17E+07	3.88E+07	1.90E+07	2.32E+07	2.71E+07
SM C26:1	61.5	841.7157	5.96E+06	7.42E+06	8.84E+06	2.79E+06	3.28E+06	3.89E+06	2.27E+06	2.68E+06	3.26E+06	1.62E+06	1.72E+06	2.10E+06
SM C26:0	62.6	843.7314	2.18E+06	2.49E+06	2.90E+06	1.35E+06	1.61E+06	1.72E+06	8.51E+05	9.54E+05	1.32E+06	7.43E+05	7.40E+05	8.38E+05
Dhsm C14:0	55.0	677.5592	4.61E+06	5.56E+06	6.88E+06	2.39E+06	3.04E+06	2.68E+06	1.25E+06	1.75E+06	2.07E+06	3.88E+06	4.57E+06	5.59E+06
dHSM C16:0	56.5	705.5905	2.02E+07	2.42E+07	2.63E+07	9.59E+06	1.20E+07	1.01E+07	7.47E+06	9.52E+06	1.08E+07	1.31E+07	1.53E+07	1.79E+07
dHSM C22:1	59.5	785.6531	8.74E+06	1.06E+07	1.27E+07	4.09E+06	5.45E+06	4.56E+06	4.63E+06	5.05E+06	5.62E+06	2.46E+06	3.46E+06	4.79E+06

Table S4. Lipid species, and mass-to-charge ratios (*m/z*'s) in each sample. Fold change was determined as [Abundance compound] / [Abundance Control] for each lipid species. The total carbon number on the acyl chains and the degree of unsaturation of the fatty acyl chains in the lipid species are listed, i.e. 16:0 corresponds to 16 carbons and 0 double bonds.

					**Compound 1**		**Econea®**	
**Species**	**Adduct**	***m/z***	**Observed mass**	**PPM error (<5)**	**FC**	**P-Value**	**FC**	**P-Value**
PC C28:3	$[M + H]^+$	672.4604	672.4618	-2.1	1.0	9.03E-01	1.7	7.91E-03
PC C30:1	$[M + H]^+$	704.5230	704.5234	-0.5	0.7	3.43E-02	2.5	9.51E-03
PC C30:0	$[M + H]^+$	706.5387	706.5382	0.7	0.9	2.36E-01	1.4	3.42E-02
PC C32:3	$[M + H]^+$	728.5230	728.5228	0.3	0.8	2.25E-01	2.9	5.12E-03
PC C32:2	$[M + H]^+$	730.5387	730.5386	0.1	0.8	7.99E-02	2.3	6.06E-03

PC C32:1	$[M + H]^+$	732.5543	732.5571	-3.8	1.0	4.45E-01	1.6	5.49E-03
PC 32:0	$[M + H]^+$	734.5700	734.5712	-1.7	0.8	4.81E-03	0.7	2.62E-03
PC C34:4	$[M + H]^+$	754.5387	754.5373	1.8	1.0	8.45E-01	1.3	1.06E-01
PC C34:3	$[M + H]^+$	756.5543	756.5558	-1.9	0.8	3.35E-02	1.8	6.09E-03
PC C34:2	$[M + H]^+$	758.5700	758.5723	-3.1	0.9	6.35E-02	1.5	4.80E-03
PC C34:1	$[M + H]^+$	760.5856	760.5882	-3.4	1.0	6.35E-01	1.0	2.74E-01
PC C34:0	$[M + H]^+$	762.6013	762.5974	5.1	1.1	1.61E-01	0.6	7.03E-04
PC C36:4	$[M + H]^+$	782.5700	782.5729	-3.7	0.9	2.29E-01	1.3	8.76E-03
PC C36:3	$[M + H]^+$	784.5856	784.5883	-3.4	0.9	1.75E-01	1.4	3.12E-03
PC C36:2	$[M + H]^+$	786.6013	786.6013	0.0	1.0	1.85E-01	1.2	1.83E-02
PC C36:1	$[M + H]^+$	788.6169	788.6195	-3.3	0.9	1.15E-01	1.0	5.95E-01
PC C38:5	$[M + H]^+$	808.5856	808.5856	0.0	0.8	1.63E-03	1.2	1.69E-02
PC C38:4	$[M + H]^+$	810.6013	810.6031	-2.2	0.8	2.15E-03	1.2	3.62E-02
PC C38:3	$[M + H]^+$	812.6169	812.6165	0.5	0.8	2.82E-03	1.0	6.17E-01
PC C38:2	$[M + H]^+$	814.6326	814.6331	-0.6	0.9	3.25E-01	1.0	7.33E-01
PC C40:6	$[M + H]^+$	834.6013	834.6004	1.1	0.7	7.72E-03	0.6	3.83E-03
PC C40:5	$[M + H]^+$	836.6169	836.6169	0.0	0.7	8.04E-04	1.0	5.44E-01
PC C40:4	$[M + H]^+$	838.6326	838.6309	2.0	0.8	2.03E-02	1.2	2.05E-01
PC C40:2	$[M + H]^+$	842.6639	842.6642	-0.4	1.0	8.39E-01	1.2	1.89E-01
PC C40:1	$[M + H]^+$	844.6795	844.6815	-2.3	1.1	2.99E-01	0.6	3.44E-04
PC C40:0	$[M + H]^+$	846.6952	846.6952	0.0	1.7	1.29E-03	1.0	9.20E-01
PC C42:6	$[M + H]^+$	862.6326	862.6294	3.7	0.6	1.05E-03	0.8	9.76E-02

PC C42:5	$[M + H]^+$	864.6482	864.6478	0.5	0.6	1.48E-03	0.9	2.58E-01
PC C42:4	$[M + H]^+$	866.6639	866.6601	4.4	0.6	6.67E-04	0.8	1.21E-01
PC C42:3	$[M + H]^+$	868.6795	868.6778	2.0	0.7	2.06E-02	0.5	3.70E-03
PC C42:2	$[M + H]^+$	870.6952	870.6958	-0.7	1.0	8.10E-01	0.5	7.84E-04
PC C42:1	$[M + H]^+$	872.7108	872.7123	-1.7	1.3	2.01E-02	0.5	1.63E-03
PC C42:0	$[M + H]^+$	874.7265	874.7265	0.0	1.8	1.47E-03	0.5	7.76E-05
PC C44:6	$[M + H]^+$	890.6639	890.6623	1.8	0.5	1.71E-03	0.6	6.28E-04
PC C44:5	$[M + H]^+$	892.6795	892.6765	3.4	0.5	9.01E-04	0.5	1.11E-04
PC C44:4	$[M + H]^+$	894.6952	894.6958	-0.7	0.5	1.10E-02	0.5	3.03E-03
PC C44:3	$[M + H]^+$	896.7108	896.7118	-1.1	0.9	3.91E-01	0.4	2.49E-03
PC C44:2	$[M + H]^+$	898.7265	898.7269	-0.4	1.1	3.34E-01	0.4	7.36E-04
PC C44:1	$[M + H]^+$	900.7421	900.7427	-0.7	1.6	1.11E-03	0.5	5.21E-04
PC C44:0	$[M + H]^+$	902.7578	902.7566	1.3	1.9	5.36E-03	0.9	6.27E-01
PC C46:6	$[M + H]^+$	918.6952	918.6926	2.8	0.6	1.89E-02	0.5	3.38E-03
PC C46:5	$[M + H]^+$	920.7108	920.7137	-3.1	0.8	1.39E-01	0.5	2.65E-03
PC C46:4	$[M + H]^+$	922.7265	922.7243	2.4	1.0	9.48E-01	0.5	5.71E-04
PC C46:3	$[M + H]^+$	924.7421	924.7384	4.0	1.1	5.60E-01	0.5	3.08E-04
PC C46:2	$[M + H]^+$	926.7578	926.7581	-0.3	1.4	1.78E-03	0.4	2.11E-03
PC C48:6	$[M + H]^+$	946.7265	946.7269	-0.4	1.6	1.29E-03	-	-
PC C48:5	$[M + H]^+$	948.7421	948.7396	2.6	0.9	1.55E-01	0.7	1.50E-02
DAG C34:2	$[M - OH]^+$	575.5039	575.5089	-0.3	1.0	9.03E-01	1.6	1.24E-02
DAG C34:1	$[M - OH]^+$	577.5196	577.5196	0.0	1.1	6.67E-01	1.2	1.66E-02
DAG C36:4	$[M - OH]^+$	599.5039	599.5052	-2.1	0.9	6.50E-01	1.4	1.75E-02

DAG C36:2	[M - OH] $^+$	603.5352	603.5354	-0.3	1.0	9.93E-01	1.4	4.96E-03
DAG C36:1	[M - OH] $^+$	605.5509	605.5514	-0.9	0.9	6.14E-01	1.0	6.70E-01
DAG C38:5	[M - OH] $^+$	625.5196	625.5197	-0.2	0.9	6.90E-01	1.1	6.94E-01
DAG C38:4	[M - OH] $^+$	627.5352	627.5368	-2.5	0.9	5.02E-01	1.1	3.98E-01
DAG C36:4	[M + H]$^+$	617.5145	617.5164	-3.1	0.9	9.39E-02	0.8	1.67E-01
DAG C36:0	[M + H]$^+$	643.5302	643.5315	-2.0	0.8	3.61E-02	1.2	1.55E-01
TAG C30:4	[M + NH4]$^+$	564.4259	564.4293	-5.4	1.3	5.64E-04	1.8	3.48E-02
TAG C38:4	[M + NH4]$^+$	676.5511	676.5475	3.5	0.7	1.02E-01	1.0	9.19E-01
TAG C48:4	[M + NH4]$^+$	816.7076	816.7051	3.1	0.7	4.18E-02	0.7	8.48E-02
TAG C50:4	[M + NH4]$^+$	848.7702	848.7759	-6.7	1.3	1.29E-01	1.0	5.79E-01
TAG C50:2	[M + NH4]$^+$	848.7707	848.7728	-2.5	1.2	6.26E-02	0.9	8.80E-02
TAG C50:1	[M + NH4]$^+$	850.7864	850.7906	-5.0	1.4	2.17E-02	0.7	9.09E-04
TAG C54:5	[M + NH4]$^+$	898.7864	898.7863	0.1	0.8	3.59E-02	1.5	1.98E-04
TAG C54:4	[M + NH4]$^+$	900.8020	900.8049	-3.2	1.2	3.39E-01	1.5	9.57E-03
TAG C54:1	[M + NH4]$^+$	906.8490	906.8473	1.8	3.0	5.50E-05	1.0	9.59E-01
TAG C54:0	[M + NH4]$^+$	908.8646	908.8656	-1.1	1.2	1.31E-01	1.0	6.64E-01
TAG C56:8	[M + NH4]$^+$	920.7707	920.7705	0.2	0.4	4.38E-04	3.0	5.86E-04
TAG C56:6	[M + NH4]$^+$	924.8020	924.8067	-5.1	0.7	1.21E-02	1.9	8.09E-04
TAG C58:8	[M + NH4]$^+$	948.8020	948.8063	-4.5	0.5	1.57E-02	3.0	1.61E-03
TAG C58:2	[M + NH4]$^+$	960.8959	960.8985	-2.7	1.7	1.83E-02	1.0	7.16E-01
TAG C60:6	[M + NH4]$^+$	980.8646	980.8694	-4.9	1.3	2.91E-02	1.0	8.60E-01
TAG C60:4	[M + NH4]$^+$	984.8959	984.8980	-2.1	1.2	7.00E-02	0.4	3.78E-03
Cer C14:0	[M - H]$^-$	508.4730	508.4741	-1.6	0.5	2.80E-04	2.4	1.26E-02

Cer C16:1	$[M - H]^-$	534.4886	534.4875	2.3	0.6	3.50E-03	1.1	7.38E-01
Cer C18:1	$[M - H]^-$	562.5199	562.5189	2.0	0.4	6.35E-05	0.3	6.21E-04
Cer C18:0	$[M - H]^-$	564.5356	564.5381	-4.7	0.4	3.05E-05	0.6	6.19E-03
Cer C20:0	$[M - H]^-$	592.5669	592.5682	-2.2	0.5	7.73E-05	0.6	2.71E-03
Cer C22:1	$[M - H]^-$	618.5825	618.5837	-1.9	0.4	7.85E-05	0.3	3.27E-04
Cer C22:0	$[M - H]^-$	620.5982	620.5995	-2.1	0.6	2.35E-04	0.5	1.97E-03
Cer C24:1	$[M - H]^-$	646.6138	646.6155	-2.6	0.6	2.64E-04	0.6	3.32E-03
Cer C24:0	$[M - H]^-$	648.6295	648.6305	-1.5	1.0	4.23E-01	0.4	3.32E-04
Cer C26:1	$[M - H]^-$	674.6451	674.6471	-3.0	0.7	1.79E-03	0.6	2.60E-03
Cer C26:0	$[M - H]^-$	676.6608	676.6645	-5.5	1.3	1.59E-02	0.4	1.62E-04
DiHCer C16:0	$[M - H]^-$	538.5199	538.521	-2.0	0.5	1.52E-06	8.9	1.36E-03
DiHCer C18:0	$[M - H]^-$	566.5512	566.5541	-5.1	0.5	1.49E-03	3.3	9.02E-04
DiHCer C20:0	$[M - H]^-$	594.5825	594.5819	1.0	0.5	2.25E-03	1.7	2.43E-04
DiHCer C22:0	$[M - H]^-$	622.6138	622.6137	0.2	0.6	7.40E-04	1.7	3.72E-04
DiHCer C24:0	$[M - H]^-$	650.6451	650.6452	-0.2	0.9	2.91E-01	0.8	2.69E-02
SM C14:0	$[M + H]^+$	675.5436	675.5443	-1.0	0.7	1.26E-01	1.4	1.65E-01
SM C16:1	$[M + H]^+$	701.5592	701.5598	-0.9	0.7	1.36E-01	1.1	7.33E-01
SM C16:0	$[M + H]^+$	703.5749	703.577	-3.0	0.8	1.97E-01	1.2	3.68E-01
SM C24:0	$[M + H]^+$	815.7001	815.703	-3.6	0.7	4.29E-02	0.7	8.67E-02
SM C18:1	$[M + H]^+$	729.5905	729.5876	4.0	0.6	1.40E-02	1.0	8.32E-01
SM C18:0	$[M + H]^+$	731.6062	731.6038	3.3	0.6	1.65E-02	0.9	4.67E-01
SM C22:0	$[M + H]^+$	787.6688	787.6676	1.5	0.5	1.53E-02	0.8	2.31E-01
SM C24:1	$[M + H]^+$	813.6844	813.6855	-1.4	0.5	8.67E-03	0.7	9.92E-02

SM C26:1	$[M + H]^+$	841.7157	841.7156	0.1	0.4	1.01E-02	0.7	4.51E-02
SM C26:0	$[M + H]^+$	843.7314	843.7291	2.7	0.6	1.46E-02	0.7	1.38E-01
dHSM C14:0	$[M + H]^+$	677.5592	677.558	1.5	0.5	1.22E-02	2.8	5.58E-03
dHSM C16:0	$[M + H]^+$	705.5905	705.590	1.0	0.4	2.56E-03	1.7	2.29E-02
dHSM C22:1	$[M + H]^+$	59.531	785.649	4.7	0.4	7.81E-03	0.8	3.27E-01

CER: ceramide; DAG: diacylglycerol; DeoxyCER: deoxyceramide; DiHCER: dihydroceramide; DiHDeoxyCER: dihydrodeoxyceramide; n.d: not detected; PC: phosphatidylcholine; SM: sphingomyelin; TAG: triacylglycerol.

Table S5. MS/MS analysis of PCs and SMs most abundant lipid species and respective most common *m/z* fragments in order of intensity TThe totalotal [2-4]. The total carbon number on the acyl chains and the degree of unsaturation of the fatty acyl chains in the lipid species are listed, i.e. 16:0 corresponds to 16 carbons and 0 double bonds.

**Lipid assigned**	***m/z* fragments**
C32:0 PC	184.0777, 124.9996, 104.1051, 166.0654
C38:4 PC	184.0758, 124.9974, 104.1034, 166.0641
C44:1 PC	184.0780, 124.9993, 104.1033, 185.0799
C18:0 SM	184.0772, 124.9979, 104.1040, 166.0633
C24:0 SM	184.0767, 124.9977, 104.1039, 166.0647
C26:1 SM	184.0777, 124.9985, 104.1027, 166.0643

3. References

1. Pereira, D.; Gonçalves, C.; Martins, B.T.; Palmeira, A.; Vasconcelos, V.; Pinto, M.; Almeida, J.R.; Correia-da-Silva, M.; Cidade, H. Flavonoid Glycosides with a Triazole Moiety for Marine Antifouling Applications: Synthesis and Biological Activity Evaluation. *Marine Drugs* **2021**, *19*, 5.
2. Brovkovych, V.; Izhar, Y.; Danes, J.M.; Dubrovskyi, O.; Sakallioglu, I.T.; Morrow, L.M.; Atilla-Gokcumen, G.E.; Frasor, J. Fatostatin induces pro- and anti-apoptotic lipid accumulation in breast cancer. *Oncogenesis* **2018**, *7*, 66, doi:10.1038/s41389-018-0076-0.

3. Li, N.; Lizardo, D.Y.; Atilla-Gokcumen, G.E. Specific Triacylglycerols Accumulate via Increased Lipogenesis During 5-FU-Induced Apoptosis. *ACS Chemical Biology* **2016**, *11*, 2583-2587, doi:10.1021/acschembio.6b00410.
4. del Solar, V.; Lizardo, Darleny Y.; Li, N.; Hurst, Jerod J.; Brais, Christopher J.; Atilla-Gokcumen, G.E. Differential Regulation of Specific Sphingolipids in Colon Cancer Cells during Staurosporine-Induced Apoptosis. *Chemistry & Biology* **2015**, *22*, 1662-1670, https://doi.org/10.1016/j.chembiol.2015.11.004.
